# A Rare Case of Aortic Valve Thrombosis in Patient with Idiopathic Hypereosinophilic Syndrome

**DOI:** 10.1155/2015/607107

**Published:** 2015-09-08

**Authors:** Elisabetta Grolla, Michele Dalla Vestra, Luca Bonanni, Ada Cutolo, Fausto Rigo

**Affiliations:** ^1^Department of Cardiology, Ospedale dell'Angelo, Mestre, 30174 Venezia, Italy; ^2^Angiology Unit, Ospedale dell'Angelo, Mestre, 30174 Venezia, Italy; ^3^Department of Internal Medicine, Ospedale dell'Angelo, Mestre, 30174 Venezia, Italy

## Abstract

Idiopathic hypereosinophilic syndrome (HES) is characterized by persistent eosinophilia and eosinophil-mediated organ-system damage. Cardiac thrombosis and thromboembolic complications represent common causes of morbidity and mortality and usually involve cardiac ventricles or mitral and prosthetic valves, while the involvement of the aortic valve is extremely rare in HES. Here we report peculiar multimodality images of an atypical case of extended thrombosis of the aortic valve, complicated by myocardial ischemia and asymptomatic cerebral ischemia, likely due to thrombus embolization, occurring in a 48-year-old man with HES. Prompt anticoagulant and steroid therapy lead to rapid and complete resolution of the thrombotic lesions, allowing preserving the native valve and preventing further embolic events.

## 1. Introduction

Idiopathic hypereosinophilic syndrome (HES) is defined as an unexplained eosinophil count greater than 1,500 cells/mL, persisting longer than 6 months, and resulting in eosinophil-mediated single- or multiple-organ damage [1, 2]. Cardiac involvement and thromboembolic complications are present in 40–50% of patients with active disease, represent major sources of morbidity, and are responsible for the observed high mortality (estimated 5–10%) [3, 4]. The typical characteristic cardiac abnormalities are endomyocardial fibrosis and ventricular thrombosis. Native and prosthetic mitral valve thrombosis are also described in HES [5], but aortic valve involvement is very unusual.

## 2. Case Presentation

**Figure 1:**
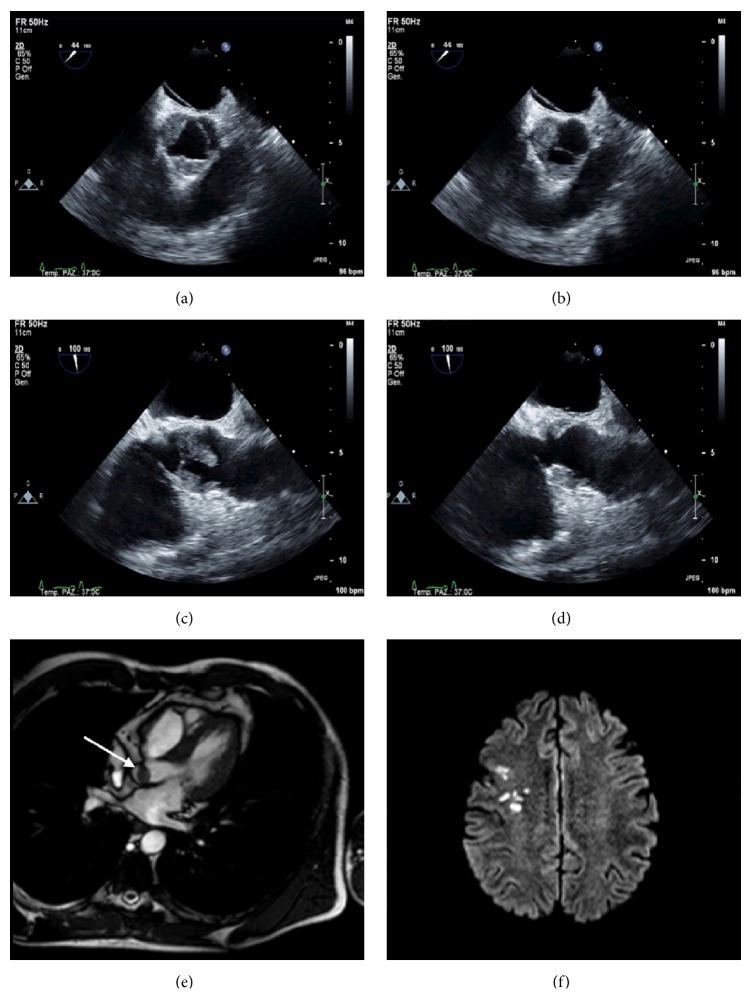
Thrombotic obliteration of the right and no-coronary Valsalva sinuses in systole (a) and diastole (b) in the upper transesophageal short axis view. Floating thrombi attached to the aortic side of the right and no-coronary Valsalva sinuses, extended to the corresponding cusps in diastole (c) and systole (d), in the upper transesophageal long axis view. (e) Cardiac MRI image showing thrombotic mass attached to the aortic valve (arrow). (f) Multiple cortical-subcortical ischemic lesions in the right frontal lobe, at the cerebral MRI.

A 48-year-old man was admitted to the department of emergency for chest pain, nausea, and vomiting with evidence of ST depression in lateral leads and increase in Troponin I levels. He was currently taking prednisone (25 mg daily) for an idiopathic hypereosinophilic syndrome (HES), diagnosed seven months before. No fever or other symptoms were observed. The patient was treated with aspirin, *β*-blockers, and heparin and underwent urgent coronary angiography that was negative. Later the patient was admitted to the department of cardiology. Laboratory tests showed leukocytosis with normal eosinophil count. Transthoracic and transesophageal echocardiography showed images suggestive of extensive thrombosis of the aortic root, with involvement and obliteration of the right coronary and the no-coronary sinuses by voluminous and floating thrombotic formations (Figures 1(a), 1(b), 1(c), and 1(d)), without significant valve dysfunction. Cardiac MRI showed normal biventricular morphology and function, confirming the thrombotic origin of the masses, since they did not enhance postcontrast imaging (Figure 1(e)). Also a cerebral MRI was performed, with demonstration of multiple cortical-sub-cortical recent ischemic lesions on the right frontal lobe, with typical features of cardioembolic lesions (Figure 1(f)). Negativity of serological testing and blood cultures together with the clinical features allowed exclusion of infective endocarditis; also autoimmune and thrombophilic evaluations were negative. The patient was immediately treated with intravenous heparin and subsequently with oral anticoagulant therapy (warfarin); second-line chemotherapy agents for HES were not started, but prednisone therapy was increased to 50 mg daily and a close clinical and echocardiographic in-hospital follow-up was programmed. Complete resolution of the thrombosis was recorded after 2 weeks and the patient remained asymptomatic and did not show further embolic events.

## 3. Discussion

HES is characterized by persistent eosinophilia and eosinophil-mediated organ-system damage. A varying spectrum of end-organ involvement can occur, but the most frequently affected systems are the bone marrow, the heart, and the nervous system [6]. Cardiac involvement in HES is common (in 40–50% of cases) and represents the prevalent cause of death in HES patients.

The cardiac pathology of HES has traditionally been divided into three stages: acute necrosis, thrombosis, and fibrosis. The acute necrosis, due to eosinophilic infiltration of the endocardium, is followed by formation of mural thrombi. The final stage is represented by endomyocardial fibrosis that leads to restrictive cardiomyopathy [6, 7].

Heart failure, intracardiac thrombus formation often involving both ventricles, the ventricular outflow tracts and the subvalvular regions, myocardial ischemia, arrhythmias, pericarditis, and syncope are the clinical manifestations of cardiac involvement.

According to literature data, valve thrombosis usually concerns mitral and prosthetic valves, while aortic involvement is very rare, especially on the vascular side of the valve. The scenario presented here is unique because it reports a rare case of massive aortic valve thrombosis attached to the vascular side of the valve and extended up to the right and no-coronary Valsalva sinuses, in the absence of significant valve dysfunction, occurring in a patient with HES.

Our patient initially showed typical signs and symptoms of myocardial ischemia. Myocardial infarction is a rarely reported complication of HES but has been described to occur as the result of an embolic event due to endomyocardial fibrosis and thrombus in the left ventricular outflow tract [8, 9]. In this case, the myocardial infarction experienced by the patient and the presence of recent, multiple, and asymptomatic cerebral ischemic lesions at MRI imaging were likely secondary to coronary and cerebral embolization from the large and floating masses attached to the aortic valve [10].

Another peculiarity of this case is represented by the fact that aortic valve thrombosis and its thromboembolic complications occurred despite a normal eosinophil count, in an apparent “dormant” phase of the disease, also suggesting that absolute eosinophil count does not always correlate with eosinophil-mediated tissue damage [11].

The prompt start of anticoagulant therapy and steroid therapy have led to rapid and complete resolution of the thrombotic lesions, allowing preserving the native valve without any significant valve dysfunction and preventing further embolic events.

## 4. Conclusion

In conclusion we reported peculiar multimodality images of a rare case of severe thrombosis of the aortic valve, complicated by myocardial ischemia and asymptomatic cerebral ischemia, likely due to thrombus embolization, occurring in a patient with HES.

Physicians should be aware of the thromboembolic complications of HES, ensuring vigilant echocardiographic follow-up of these patients, indeed the rapid recognition of a valvular involvement, and the timely start of effective therapy dramatically increase the possibility of preserving the native valve and can reduce the incidence of embolic events.
